# Tapia Syndrome Secondary to Hypoglossal Nerve Schwannoma: A Case Report on a Rare Medical Condition

**DOI:** 10.7759/cureus.98441

**Published:** 2025-12-04

**Authors:** Anwar Khan, David Gorelov, Brittani P Kongala, William Crook, John T Lanza

**Affiliations:** 1 Otolaryngology - Head and Neck Surgery, University of South Florida, Tampa, USA; 2 General Surgery, The Jewish Hospital, Cincinnati, USA; 3 Medicine, Florida State University College of Medicine, Tallahassee, USA; 4 Radiation Oncology, HCA Florida Lawnwood Hospital, Fort Pierce, USA; 5 Otolaryngology, ENT Allergy and Associates, Fort Pierce, USA

**Keywords:** hypoglossal nerve palsy, recurrent laryngeal nerve palsy, schwannoma, stereotactic body radiation therapy, tapia syndrome, vagus nerve palsy

## Abstract

Tapia syndrome (TS) is the ipsilateral palsy of the hypoglossal nerve (CN XII) and the recurrent laryngeal branch of the vagus nerve (CN X). It typically presents with tongue deviation toward the affected nerve, dysphagia, and dysphonia due to unilateral paralysis of the intrinsic tongue muscles and vocal cords. Common etiologies include orotracheal intubation or direct trauma, with nontraumatic peripheral causes rarely reported. We present the case of a 77-year-old woman with progressive hoarseness, dysphagia, and right tongue deviation. She reported no history of intubation, recent surgery, or trauma. Physical examination revealed right tongue atrophy, and rigid laryngoscopy showed bowing of the right vocal fold, severely decreased mobility of the right true vocal cord, and moderate phase asymmetry. MRI imaging identified a 3.2 cm x 1.7 cm mass near the hypoglossal canal extending into the right neck, consistent with a schwannoma compressing CN X and CN XII. The patient was treated with stereotactic body radiation therapy (SBRT), which was well-tolerated. On follow-up, 18 months later, her symptoms remained stable with mild improvement in hoarseness and no progression of the schwannoma on imaging. This case illustrates a rare central cause of TS and discusses the benefits of SBRT when surgery poses high risk.

## Introduction

First described by the Spanish otolaryngologist Antonio Garcia Tapia in 1904, Tapia syndrome (TS) is characterized by unilateral paralysis of the hypoglossal nerve (CN XII) and the ipsilateral laryngeal branches of the vagus nerve (CN X) [1,2]. Early recognition of TS is crucial, as delayed diagnosis can result in persistent nerve deficits and impaired swallowing or speech [3]. Clinically, patients present with tongue deviation toward the affected nerve, dysphonia, dysarthria, and dysphagia due to paralysis of the ipsilateral intrinsic muscles of the tongue and vocal cords [1-4].

The etiologies of TS are broadly classified as peripheral or central. Peripheral lesions (extracranial) are most common and arise from the lateral wall of the inferior portion of the oropharynx at the base of the tongue and the superior portion of the hypopharynx, where CN X and CN XII lie in close proximity [3]. Peripheral TS most often occurs secondary to orotracheal intubation in a hyperextended neck, resulting in mechanical damage or stretching to both CN X and CN XII along their respective pathways [3]. Other reported causes include neurofibromatosis of CN X and CN XII, carotid artery dissection of the ascending pharyngeal artery, and direct trauma [4]. More recently, cases of COVID-19 causing peripheral lesions of TS have been described, perhaps due to the increase in prolonged intubations [5].

In contrast, central etiologies of TS are extremely rare and involve lesions to the brainstem CN XII nucleus, nucleus ambiguus, and the pyramidal tract [2,3]. Such cases may result from malignancy, hemorrhage, ischemia, or other space-occupying lesions [2]. However, TS secondary to a peripheral nerve tumor is exceedingly rare. We present the case of an elderly woman with TS arising from a hypoglossal nerve schwannoma. Because tumor-associated TS is rarely reported, this case adds meaningful clinical insight to an underrepresented etiology.

## Case presentation

A 77-year-old woman with a history of type II diabetes, hypothyroidism, and cardiovascular disease presented to the otolaryngology clinic with progressively worsening hoarseness for the past two months. She also reported dysphagia to solids and liquids, throat pain, dry mouth, right-sided ear pain, and globus sensation. She denied any odynophagia, weight loss, or fever. Her past medical history was negative for squamous cell carcinoma of the head and neck, thyroid cancer, or excessive vocal cord use. She had no history of recent surgeries, intubations, or COVID-19 infection.

**Figure 1:**
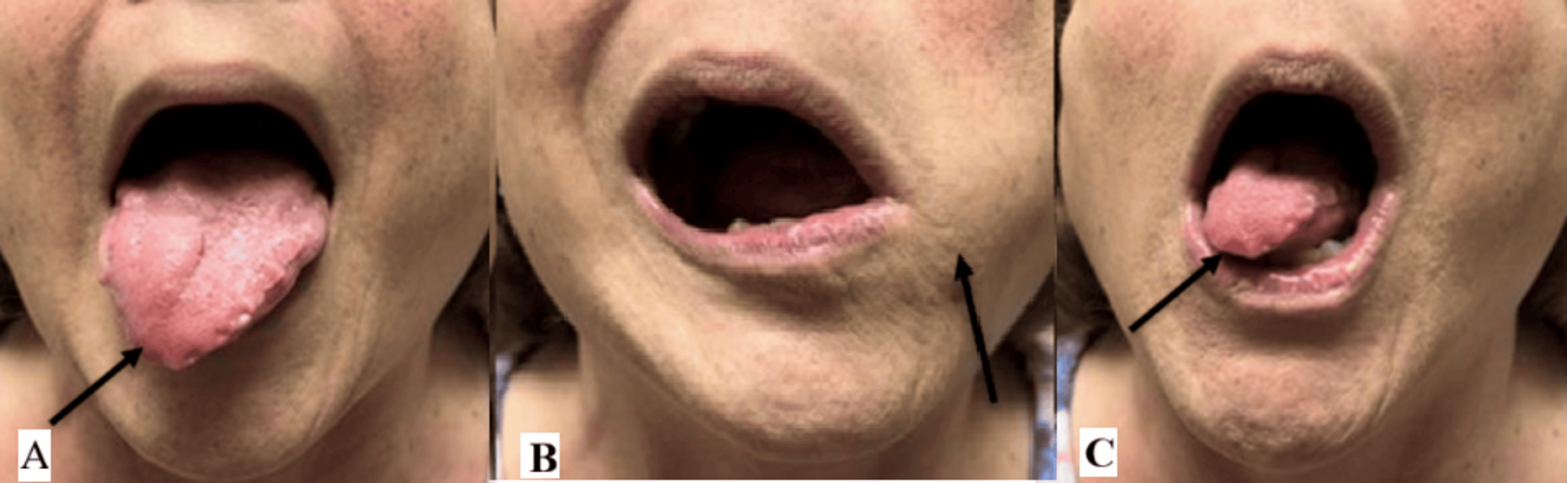
(A) Patient asked to stick the tongue outside of mouth. (B) Patient asked to stick tongue in left check. (C) Patient asked to stick tongue in right cheek

**Figure 2:**
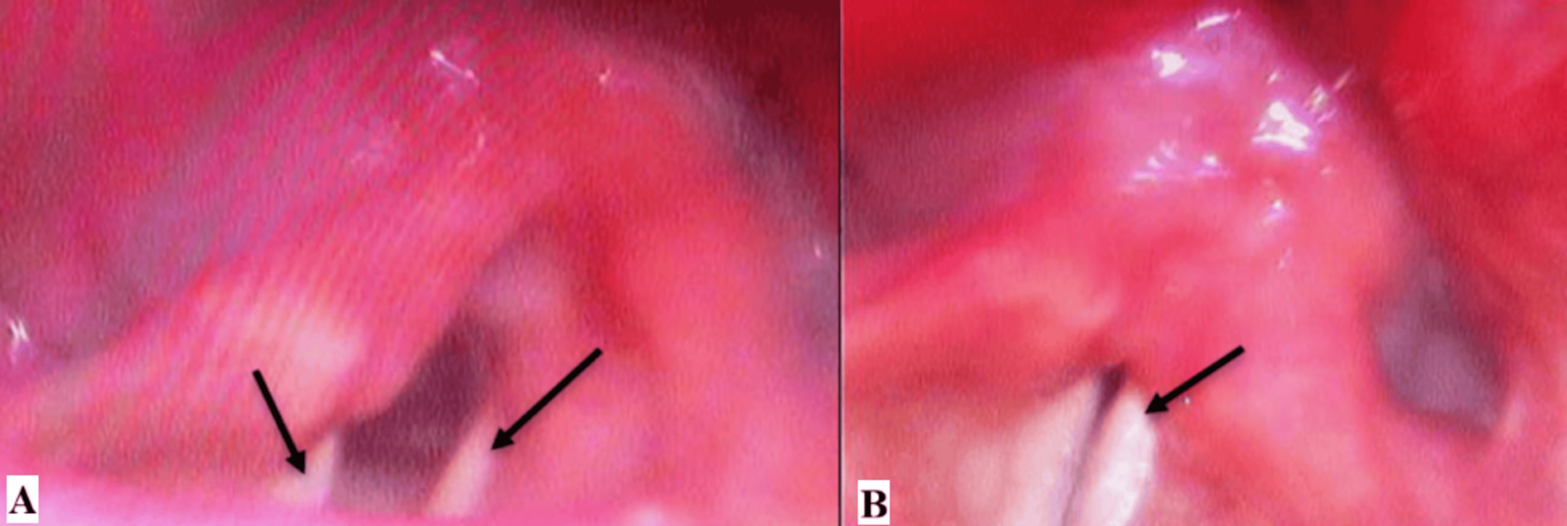
(A) Rigid laryngoscopy of vocal cord at rest. (B) Rigid laryngoscopy of vocal cord after patient instructed to say the letter “E”

**Figure 3:**
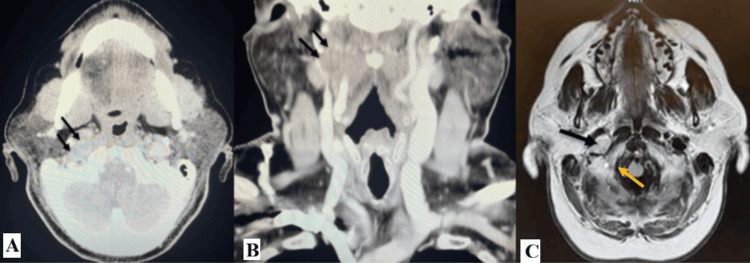
(A) An axial CT image of the head and neck showing an enlarged, well defined, hypoechoic mass. (B) Coronal view of CT imaging showing the same hypoechoic mass. (C) An axial MRI of the brain revealed a 3.2 cm × 1.7 cm mass located near the hypoglossal canal extending into the right neck. The yellow arrow indicates the medial opening of the hypoglossal canal

On physical exam, the patient’s voice was hoarse and raspy without signs of respiratory distress. Examination of the ears, nose, head, and neck was unremarkable with no palpable masses or nodules. Oral cavity inspection revealed right-sided tongue atrophy with deviation of the tongue toward the right. Images of the physical exam findings are visualized in Figure 1. A flexible laryngoscopy, seen in Figure 2, demonstrated bowing of the right vocal fold free edge, severely decreased mobility of the right true vocal cord, and moderate phase asymmetry. CT of the head and neck showed an enlarged, well-defined, hypoechoic mass. A subsequent MRI confirmed a 3.2 cm × 1.7 cm lesion located near the hypoglossal canal extending into the right neck, consistent with TS secondary to a tumor. CT and MRI findings are shown in Figure 3. The patient was diagnosed with a benign peripheral nerve schwannoma of the right CN XII with subsequent compression of CN X.

Treatment options in this case were complicated due to the location of the schwannoma and proximity to critical neurovascular structures. The patient was concerned about surgical intervention due to the high risk of surgery. Therefore, stereotactic body radiation therapy (SBRT) was deemed to be the most appropriate management strategy. SBRT delivers high-dose, conformal radiation precisely to the target lesion while sparing surrounding tissue. The patient received a total of 18 Gray (Gy) delivered in three equal treatments of 6 Gy each from October 11, 2023, to October 19, 2023. She tolerated the treatment well with no toxic effects.

**Figure 4:**
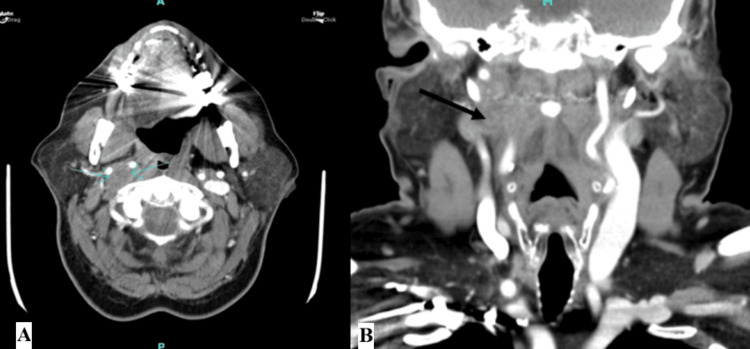
(A) Axial CT showing a defined hypoechoic mass after treatment with SBRT. (B) Coronal CT showing the same lesion without further progression from initial imaging pretreatment

In subsequent follow-up encounters, the patient reported mild improvement in hoarseness but no other improvement from her baseline evaluation. However, her symptoms had not progressed after treatment, and she did not endorse any dysphagia or new symptoms. No additional cranial nerve deficits were noted. She was referred to speech therapy for further improvement of her symptoms, but it is unknown if she participated in therapy. Follow-up CT imaging of the neck in April 2025, 18 months after her diagnosis and treatment with SBRT, demonstrated no progression of the schwannoma. CT is shown in Figure 4.

## Discussion

TS is a diagnosis of exclusion that relies heavily on clinical evaluation [2]. In this patient, the absence of common risk factors, such as no recent history of orotracheal intubations, surgery, direct trauma, or malignancy, meant that the diagnosis of TS depended primarily on physical exam findings. The atrophied and deviated tongue indicated palsy of CN XII, while the right vocal fold bowing and immobility observed on laryngoscopy reflected a lesion to CN X. Because central causes of TS are exceedingly rare, these physical exam findings raised suspicion for a space-occupying lesion. As such, TS should always remain a differential diagnosis in any patient presenting with classic symptoms, regardless of the presence or absence of typical risk factors.

The anatomy of the craniocervical junction is complex, with many cranial nerves traveling in proximity to one another. CN X and CN XII converge near the hypoglossal canal and its junction with the carotid sheath. CN XII exits the hypoglossal canal medial to the internal carotid artery and internal jugular vein, then descends posterior to CN X within the carotid sheath [6]. An anastomosis between CN X and CN XII occurs near the nodose ganglion of CN X [6]. A lesion in this region can therefore produce simultaneous palsies of both nerves through mass effect. This anatomic relationship corresponds with the imaging findings in our patient and is the most likely location of the schwannoma. TS is differentiated from other jugular foramen syndromes, such as Vernet, Collet-Sicard, and Villaret, in the absence of involvement of the accessory nerve (CN XI) [3].

Most cases of TS arise from well-documented traumatic or iatrogenic etiologies, particularly postintubation injuries, where risk factors include duration of intubation, head and neck position, and upper limb manipulation above the head [2,7-10]. However, solitary cranial nerve neoplasms are rare and not well-documented, especially in the absence of predisposing genetic conditions. Only one previous case of TS secondary to a schwannoma at the craniocervical junction has been reported [7]. In that case, the lesion involved both CN X and CN XII, with the origin of the tumor impossible to determine [7]. In our case, the tumor’s proximity to the hypoglossal canal and its radiographic trajectory suggested CN XII origin, although contribution from CN X cannot be excluded.

Emerging etiologies of TS, such as viral infections, have also been described in patients without clear risk factors. During the COVID-19 pandemic, the incidence of TS rose significantly, although this is most likely due to the surge in prolonged intubations [8-10]. Infection with the Epstein-Barr virus (EBV) has also been speculated as a potential risk factor [11-13]. For instance, EBV is associated with cranial nerve palsies involving the recurrent laryngeal nerve branch of CN X and CN XII, though this is rare [11-13]. The mechanism underlying EBV-associated cranial neuropathy remains unclear. However, potential mechanisms include immune-mediated injury, subsequent denervation, and direct viral injury, which are usually more prevalent in children and adolescents [14].

The current standard of care for TS is supportive with a focus on rehabilitation through speech and language therapy, especially for peripheral etiologies of TS [2]. In 2010, Boğa and Aktas proposed a graded score for TS based on the severity of the disease, with grade I as mild, grade II as moderate, and grade III as severe [15]. They advocated for corticosteroids as a key part of treatment, proposing that steroid therapy reduces nerve swelling and may enhance recovery, but this has not been substantiated and remains controversial [15]. In addition, the proposed grading system has limited practical utility as prognosis and management are similar across severities.

Recovery outcomes of peripheral TS vary widely and depend on the underlying etiology. In a review of 23 cases of peripherally induced TS managed with varying treatments ranging from no treatment at all to steroids and nasogastric tubes, seven patients (30.4%) achieved complete functional recovery, whereas nine patients (39.1%) achieved incomplete recovery, and six patients (26.1%) achieved no recovery [3]. The general consensus is that early rehabilitation is essential for the best outcome, as recovery can take months to occur, if at all. However, the recovery and management of patients with an underlying viral etiology is still uncertain and should be further studied.

The treatment and prognosis of central TS remain more complex than those of peripheral lesions. In our patient, SBRT was selected due to the tumor’s deep-seated location near critical neurovascular structures and the high morbidity associated with surgical resection. SBRT has demonstrated excellent tumor control and symptom improvement in spinal schwannomas, which are benign peripheral nerve sheath tumors. In a Stanford series of 47 spinal schwannomas treated with CyberKnife-based SBRT (average dose 18.7 Gy), local control was 98% at a median follow-up of 29 months, with nearly half of patients exhibiting radiographic regression and over 50% reporting pain relief [16]. In addition, no cases of late radiation-induced spinal cord toxicity were observed [16]. These findings align with broader reports showing >90% local control across SBRT-treated schwannomas in the spine and skull base, with high rates of symptomatic improvement and preservation of nerve function [17]. While most data involve spinal and cranial nerve schwannomas, this evidence supports the expanding role of SBRT as a minimally invasive, function-preserving option for patients with symptomatic or inoperable peripheral nerve schwannomas.

## Conclusions

TS is characterized by the unilateral paralysis of CN X and CN XII and most commonly occurs following postsurgical intubation. In this patient case, the patient presented with classic clinical features of TS but without any of the known risk factors typically associated with TS. Initial imaging of the head and neck revealed a mass at the craniocervical junction near the intersection of CN X and CN XII, consistent with a schwannoma of CN XII. The patient underwent treatment with three rounds of SBRT with mild clinical improvement and stable disease on follow-up imaging 18 months later. TS secondary to a schwannoma in this location is exceedingly rare, and further studies are needed to clarify its incidence, pathophysiology, and optimal management. As the incidence of TS continues to rise, clinicians should maintain a high index of suspicion for TS and consider structural lesions in the differential diagnosis for patients presenting with concurrent CN X and CN XII palsies.
